# Effect of Heat Treatment on the Microstructure and Mechanical Properties of Additive Manufactured Ti-6.5Al-2Zr-1Mo-1V Alloy

**DOI:** 10.3390/ma16010160

**Published:** 2022-12-24

**Authors:** Sheng Zhang, Yuqi Zhang, Jinshun Qi, Zhiyi Zou, Yuanhong Qian

**Affiliations:** 1 School of Mechanical Engineering, University of Science and Technology Beijing, Beijing 100089, China; 2 Shunde Graduate School, University of Science and Technology Beijing, Foshan 528300, China; 3 Centre for Additive Manufacturing, University of Nottingham, Nottingham NG8 1BB, UK; 4 Beijing Xinghang Electromechanical Equipment Co., Ltd., Beijing 100074, China

**Keywords:** laser powder bed fusion, TA15 alloy, heat treatment, microstructure, mechanical property

## Abstract

Ti-6.5Al-2Zr-1Mo-1V (TA15), widely used in the aerospace industry, is a medium- to high-strength, near-α titanium alloy with high aluminium equivalent value. The TA15 fabricated via laser powder bed fusion (L-PBF) normally presents a typical brittle appearance in as-built status, with high strength and low ductility. In this study, the microstructure and properties of L-PBF TA15 were engineered by various heat treatments below the β-transus temperature (1022 °C). After heat treatment, the original acicular martensite gradually transforms into a typical lamellar α + β dual-phase structure. Withannealing temperature increases, the lamellar α phase thickened with a decreased aspect ratio. Globularisation of the α grain can be noticed when annealing above 800 °C, which leads to a balance between strength and ductility. After heat treatment between 800–900 °C, the desired combination of strength and ductility can be achieved, with elongation of about 12.5% and ultimate tensile strength of about 1100 Mpa.

## 1. Introduction

Thanks to high specific strength, excellent corrosion resistance and good biocompatibility, titanium and its alloys have been widely applied in the biomedical, automobile and aerospace industries [1]. Laser powder bed fusion (L-PBF) has been widely accepted as a promising technique for producing titanium alloy, as it can provide an excellent buy-to-fly ratio, high design freedom and the possibility of near-net-shape manufacturing [2,3,4,5,6].

L-PBF of Titanium and its alloys, particularly Ti-6Al-4V, has been extensively studied over the past decade [4,5,6,7,8]. Research reveals that high cooling rates and large temperature gradients during the L-PBF process often lead to the formation of fine acicular martensite in the as-built microstructure, with high residual stresses, which result in high strength and low ductility compared to their forging or casting counterparts [4,8]. Therefore, post-process heat treatments are often applied to modify the obtained microstructure and the corresponding mechanical properties [9,10,11,12]. For heat treatment conducted at a temperature lower than the β transus of the material, the annealing temperature is often considered to be the most important factor affecting the outcome of the heat treatment [9]. Zhang et al. [11] point out that compared to the cooling rate, the soaking temperature has a more significant effect on the ductility and strength of L-PBF Ti-6Al-4V, and the optimal mechanical properties can be obtained at an annealing temperature of 850 °C or 900 °C. Vrancken et al. [9] report that the as-built α′ martensite phase in L-PBF Ti-6Al-4V, once heat-treated to temperatures above the martensite start temperature but below the β-transus temperature, can decompose to a lamellar mixture of α and β. In their study, the best ductility (12.84 ± 1.36%) is achieved after heat treatment at 850 °C for 2 h, followed by furnace cooling. Cao et al. [8] obtain a strong and ductile L-PBF Ti-6Al-4V, with ductility similar to wrought Ti-6Al-4V, after 6 h of annealing at 800 °C. This heat treatment leads to complete decomposition of the as-built martensite, resulting in a balanced α and β lamellar microstructure. Therefore, 800 °C to 900 °C is widely considered the optimal heat treatment temperature for L-PBF Ti-6Al-4V. Furthermore, these reports undoubtedly indicate the benefit of applying heat treatment on L-PBF titanium alloy.

Ti-6Al-4V and Ti-6Al-2Zr-1Mo-1V (TA15) are backbone titanium alloys often used in modern aircraft structures [7,8,13,14]. TA15 alloy is a near-α-titanium alloy with high aluminium equivalent value, which utilises the solid solution strengthening of α-stabilising element Al to achieve high strength. In addition, the neutral element, Zr, and β-stabilising elements, Mo and V, are added to improve the process performance [15]. Therefore, TA15 has good thermal strength and weldability close to typical α titanium alloy, and can also provide decent process ductility similar to (α + β) titanium alloy. Compared to Ti-6Al-4V, TA15 alloy has a decent strength at medium to high working temperatures (400 °C to 500 °C). As the result, while Ti-6Al-4V is commonly used as a structural material in the aerospace industry, TA15 is widely applied to the load-bearing components in aircraft and engines, which operate at high temperatures for extended periods [16]. Lately, L-PBF has been successfully adopted to process TA15 alloy [17,18]. Wu et al. [19] report that TA15 produced via L-PBF could exhibit excellent tensile strength both at room and elevated temperature, due to grain refinement and nano-scale twins. Jiang et al. [20] indicate that the samples annealed at 800 °C exhibited significantly improved mechanical properties compared to as-built status. Wu et al. [13] achieve lamellar α + β microstructure and superior mechanical properties (high strength–ductility matching) after annealing L-PBF TA15 at 940 °C.

The mechanical properties of titanium alloys are mainly determined by their microstructure, and heat treatment plays a critical role in controlling the outcome [11]. As of today, post-process heat treatment for L-PBF Ti-6Al-4V has been advanced from conventional single heat treatment to alternative tailored heat treatments, such as multiple annealing [21], thermal cycling [22] and rapid heat treatment [5]. However, to our knowledge, the current research on the post-process heat treatment of L-PBF TA15 is still in its early stages, focusing on conventional single heat treatment. Moreover, the heat treatments that have been studied lack an understanding of the corresponding microstructure evolution, and the heat treatment temperatures tested are arbitrarily, instead of systematic design and selected, which limits the development of more complex multiple heat treatments. A more systematic and detailed study of the relationship between heat treatment, microstructure and mechanical properties can provide relevant information for future optimisation of heat treatment. To bridge this gap, this paper will study the microstructural features and corresponding mechanical performance of heat-treated L-PBF TA15, as well as the mechanism leading to the microstructure evolution.

## 2. Method and Material

### 2.1. L-PBF Process and Heat Treatment

The material used in this study was spherical TA15 powder with a median particle size of 33.57 um, provided by AMC (Beijing AMC Powder Metallurgy Technology Co., Ltd., Beijing, China). Cylindrical samples with a diameter of 12 mm and a height of 80 mm were built along the vertical direction via an EOS 280 L-PBF machine using the following optimised parameters: laser power of 360 W, laser scanning speed of 1200 mm/s, scan line hatch spacing of 120 μm and powder layer thickness of 30 μm. The build strategy applied has been detailed in the previous work [13].

The composition of the printed material was determined by Energy Dispersive Spectroscopy (EDS, JEOL, Tokyo, Japan, JSM-7900F) and reported in Table 1. To accurately define the β-transus of the investigated alloy, differential scanning calorimetry (DSC, TA Instruments, New Castle, Germany, SDT-Q600) was performed by heating the as-built sample to 1100 °C at a heating rate of 10 °C/min, followed by furnace cooling to room temperature. Based on critical temperatures obtained from DSC analysis and literature [17,20], six different temperatures, spread in three representative temperature ranges, were chosen as heat treatment temperatures investigated in this study (Table 2). A heating rate of 10 °C/min was applied for all the conducted heat treatments, followed by air cooling (AC). A soaking duration of 2 h, as a typical heat treatment duration for dual-phase Ti alloys [23], was chosen to achieve uniform heating of the specimens and to exclude the influence of different soaking duration on the obtained microstructure and mechanical properties.

### 2.2. Microstructure Characterisation

In this study, an inverted optical microscope (OM, Keyence, Osaka, Japan, VHX-5000) and scanning electron microscope (SEM, JEOL, Tokyo, Japan, JSM-IT100) were used to conduct microstructural analysis. X-ray diffraction analysis (Bruker, Billerica, MA, USA, D8 Advance) was applied to determine the microstructural constituents. The obtained XRD pattern was analysed via Jade6.

### 2.3. Mechanical Characterisation

Tensile tests conforming to ISO 6892-1:2009 were performed on as-built and heat-treated samples at room temperature, with a strain rate of 0.00025 s^−1^ (before yielding) and 0.0067 s^−1^ (after yielding), via an MTS CMT5205 tensile testing machine. Hardness tests were conducted using an EM-1500L Vickers microhardness tester via an indentation load of 200 gf and a dwell time of 10 s. Ten indentations were made on each condition.

## 3. Results

### 3.1. Differential Scanning Calorimetry Analysis

As a near-α titanium alloy, the low-temperature α phase → high-temperature β phase transformation of TA15 is a continuous process [24]. The minimum temperature to maintain the entire material in a full β condition is known as the β-transus temperature. When TA15 is equilibrium cooled from the single β region to room temperature, the TA15 will consist of an α phase dominant microstructure with only limited β phase retained. However, when the applied cooling rate is higher than 20 °C/s, the metastable transformation will be activated [25]. The commonly seen metastable phase for TA15 in L-PBF condition is the metastable α′ phase.

**Figure 1:**
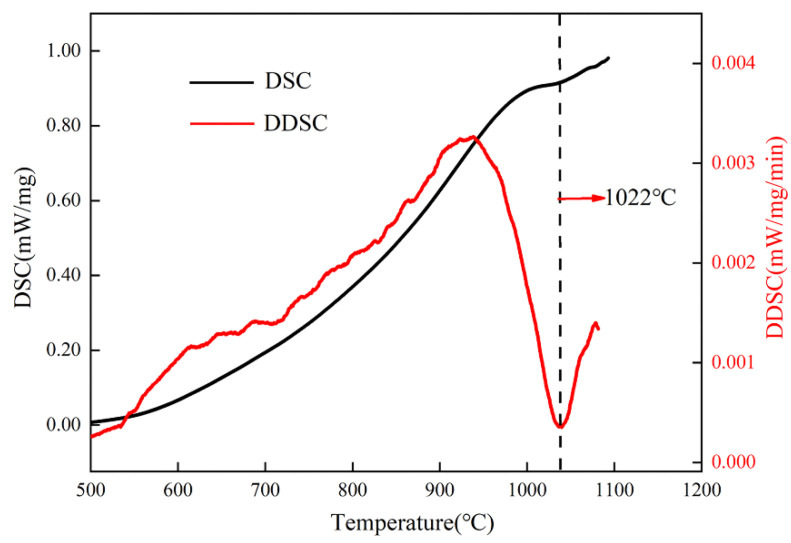
The measured DSC/DDSC curve of the as-built TA15 sample.

Figure 1 shows the measured DSC curve of the as-built TA15 sample. The thermal effect of this curve is very fragile, with no obvious endotherm or exothermic peak, the completion of the α→β phase transus is only evidenced by a baseline shift, and the migration of baseline is identified at a negligible level of around 30 °C. In order to better determine the β-phase transus temperature, the derivative of the DSC curve (DDSC) is taken to observe the variation in thermal effect. It can be noticed that the curve presents an endothermic peak that starts at approximately 700 °C, which can be attributed to the decomposition of α′→α as suggested by previous research [8,26,27]. With the increase of temperature, the material shows an endothermic peak ended at 1022 °C, which can be ascribed as the completion of the α→β phase transition. Therefore, it can be defined that the as-built TA15 samples used in this study exhibited a β-transus temperature of 1022 °C. This measured β-transus temperature differs from the values reported on the traditional manufactured TA15 (around 990 °C) [28]. The observed increase of the β-transus can be attributed to the elemental evaporation in the melt pool during the L-PBF process. Such elemental evaporation has also been noticed in other L-PBF Ti alloys [29].

### 3.2. Microstructural Constituents

**Figure 2:**
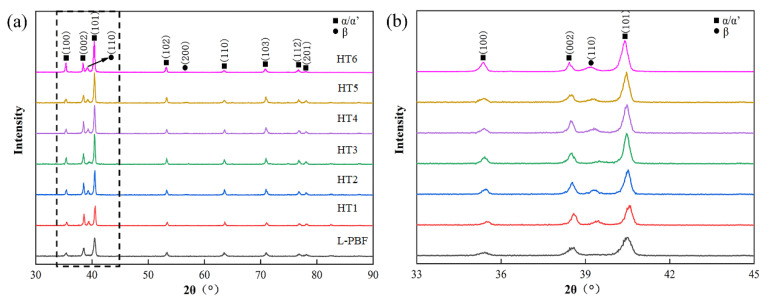
(**a**) XRD pattern of the L-PBF TA15 before and after annealing, (**b**) details within the black dotted lines in (**a**).

Figure 2 shows the measured XRD pattern of L-PBF TA15 before and after annealing, in which the majority of diffraction peaks are related to the α/α′ phase. Since both α and α′ phases are hexagonal close-packed (HCP) structures with similar lattice parameters, they share similar XRD patterns [20]. Meanwhile, a small volume fraction of the β phase appears after heat treatment, which indicates that the original α′ phase has decomposed into α + β phase. Figure 2b demonstrates that the diffraction peak positions of the as-built sample are slightly different from the annealed specimens, which can be attributed to the smaller lattice parameter of α′ martensite compared to the α phase. This is because the atomic radius of aluminium (0.143 nm) is higher than that of vanadium (0.132 nm) and molybdenum (0.136 nm), and there is a higher concentration of aluminium appears in the α phase compared to α′ martensite [30]. This will result in the shifting of diffraction peak of αʹ to a higher angle [31].

Moreover, as shown in Table 3, the full width at half maximum (FWHM) of the α/α′ peak (2θ position of 40.5°) in the as-built specimen is larger than other annealed conditions. FWHM measurement can provide information on grain distortion, dislocation density and residual stresses [32]. The difference between FWHM in the as-built and annealed conditions is because that the as-built material contains a large number of lattice defects and high residual stress due to the complex thermal history in the L-PBF process [33]. It can be noticed that the FHWM achieved a balanced value after annealing at 750 °C, which indicates that the most residual stress was eliminated at such a condition. Such finding corresponds well to other research [34]. Table 3 shows that the lattice constant of the β phase is roughly in the range of 3.221 Å to 3.241 Å, with different annealing temperatures. This is mainly caused by the diffusion of V and Mo atoms into the β phase during heat treatment, resulting in a larger lattice parameter.

### 3.3. Microstructure

#### 3.3.1. Microstructure of the As-Built TA15 Alloy

**Figure 3:**
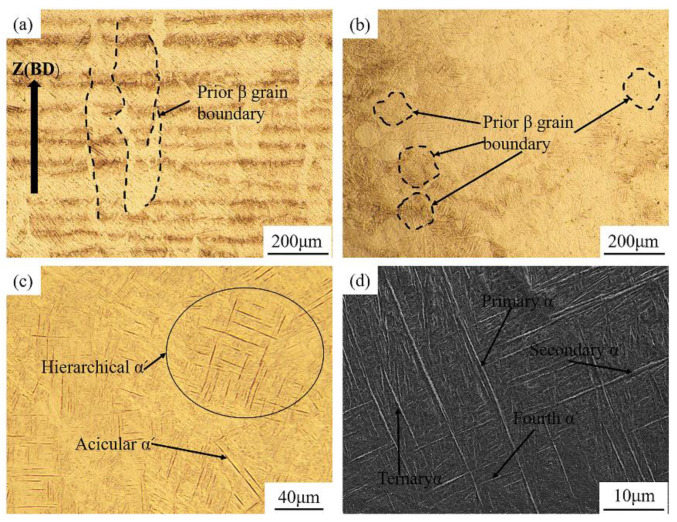
Microstructure of the as-built TA15: (**a**) OM micrograph of the vertical plane, (**b**) OM micrograph of the horizontal plane, (**c**) higher magnification of OM micrograph and (**d**) SEM micrograph of the vertical plane.

Figure 3 shows the typical microstructure of the as-built TA15 material. Near full dense specimens have been successfully obtained thanks to the employment of the optimised process parameters. In Figure 3a, columnar prior-β grains with a width of about 100 μm can be clearly observed, which is attributed to the homoepitaxial growth of the β grains during the L-PBF process. Since the laser will remelt some previously formed prior-β grains during layer-wise scanning, grains elongated along the thermal gradient direction will be formed [35]. Figure 3c shows the as-built material from the side view under higher magnification, showing that these columnar prior-β grains contain large quantities of acicular martensitic α′ phase due to the steep temperature gradient and high cooling rate during the L-PBF process [36]. As illustrated in Figure 3b, owing to the distinct scanning strategy with a rotation angle of 67°, most prior-β grains present an irregular shape on the horizontal plane. The acicular α′ phase with hierarchical structure features at different length scales is marked in Figure 3d. These interlaced acicular α′ are oriented at 30°, 60° and 90°, owning to the Burgers orientation relationship between the prior body-centred cubic β and the hexagonal close-packed α phase [37]. Concurrently, these fine martensitic α′ can be classified as primary, secondary, ternary and quartic α′ according to the size feature. Similar findings are also being reported in other research, which is correlated to multiple thermal cycles the material experienced during the L-PBF process [27].

#### 3.3.2. Microstructure Evolution during Heat Treatment

**Figure 4:**
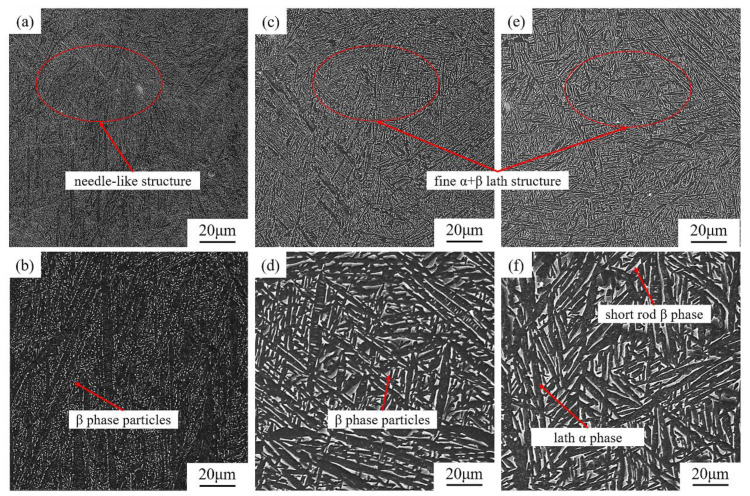
SEM micrographs of heat-treated as-built TA15; (**a**) 630 °C, (**c**) 750 °C and (**e**) 800 °C for 2 h; (**b**,**d**,**f**) show higher magnification of the micrographs.

**Figure 5:**
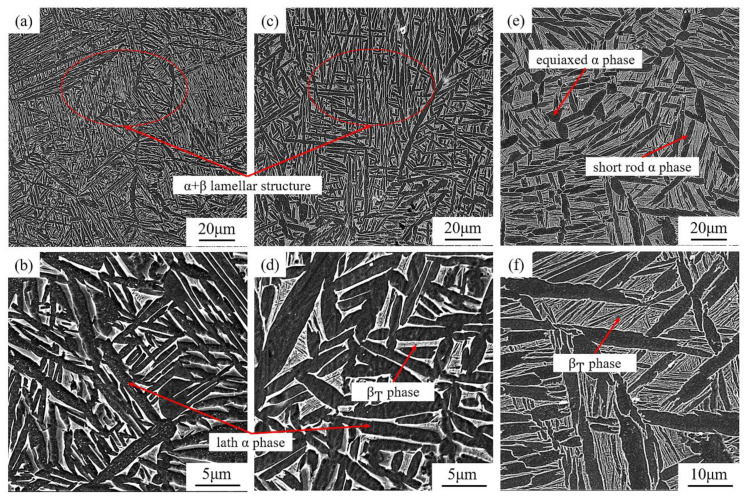
SEM micrographs of L-PBF TA15 heat-treated at (**a**) 850 °C, (**c**) 900 °Cand (**e**) 950 °C for 2 h; (**b**,**d**,**f**) show higher magnification of the micrographs.

The starting microstructure of a material is a critical factor for the microstructure’s evolution. In this study, the as-built L-PBF TA15 is filled with fine acicular α′ martensite, a metastable hcp structure containing a high density of lattice defects, such as dislocations and twins [38]. Therefore, it is reasonable to believe that under subsequent near-equilibrium heat treatment, such martensite structures will decompose to more stable final microstructures. A series of microstructures obtained after heat treatments are shown in Figure 4 and Figure 5. After heating to 630 °C and holding for 2 h (HT1), which is often called stress relief annealing, a certain portion of the acicular α′ starts to decompose into a fine lamellar α plate and white β particles, and the latter can often be found along the fine α phase boundaries (Figure 4a,b).

When the heating temperature increases to 750 °C (HT2), it is difficult to observe any trace of the original acicular martensite in the microstructure, the fine lamellar α is thickened, and the volume fraction of the β phase increases. As the heating temperature increases to 800 °C (HT3), the lamellar α becomes noticeably coarser, and the particle-like β phase transforms into a short rod shape, forming the typical lamellar α + β phases (Figure 4c–f). Following the increasing of soaking temperature, further coarsening of the α and β can be observed in the HT4 condition (Figure 5a,b). However, the morphology of the microstructure did not present a substantial variation. Owing to the high cooling rate, the transition from bcc phase (β) to hcp phase (α′) in the L-PBF process is a diffusionless process [20]. Therefore, β stabilising elements (Mo and V) are supersaturated in the as-built martensite α′. During heat treatment, the α phase nucleated at the boundary of the acicular α′ phase, and the β stabiliser will then be pushed to the region between the lamellar α phase. The newly formed β stabiliser-enriched area will be the dominant nucleation site of the β phase [39]. The latest research reveals that some β phase can also be nucleated from the lattice defects located inside the hcp (α/α′) structure [36].

When the soaking temperature reaches 900 °C (HT5), transformed β matrix (β_T_) starts to be observed in the material, which is composed of β phases and fine α phase precipitated from the matrix (Figure 5c,d). As the soaking temperature approaches β-transus, in the HT6 sample (see Figure 5e,f), a large number of secondary α phases formed in the β_T_ phase. Furthermore, with increasing heating temperature, the growth-inhibiting effect becomes weaker, and lamellar grains start to coarsen, a phenomenon driven by the reduction of interfacial energy [13].

With the heat treatment temperature increase, the lamella thickness gradually increases, and the volume fraction of the β phase shares the same trend. When the heating temperature reaches 900 °C, the growth rate of the β-phase volume fraction increases significantly. It is widely accepted that for any sub-transus heat treatment, the soaking temperature has a decisive role in controlling the size of the microstructure feature [7,13,20].

**Figure 6:**
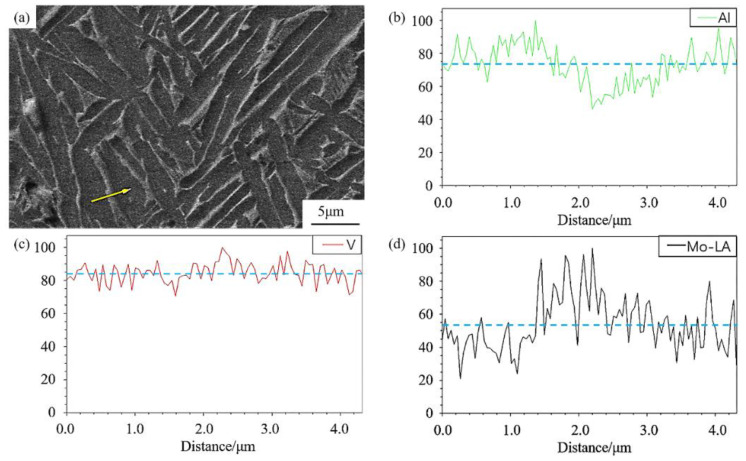
The EDS element line scan in the material heat-treated at 900 °C/2 h/AC: (**a**) SEM images of the annealed α + β microstructure, where the yellow arrow represent the EDS line scan location. (**b**–**d**) EDS line scan of Al, V and Mo.

Figure 6 shows the EDS line scan of the sample after annealing at 900 °C. It can be noticed that heat treatment will result in element segregation. The content of Al, Mo and V elements varies significantly across the α/β boundary, as shown in Figure 6b–d. Since V and Mo are β-phase-stabilising elements, two elements are enriched in the β phase; while Al, as the α-stabilising element, mainly concentrates in the α phase [22]. According to the EDS results, the Al-enriched area has a dark appearance, and the V- and Mo-enriched areas appear to be brighter, which further suggests that the bright area in SEM images is the β phase.

As a supersaturated solid solution, acicular martensite contains significant β-phase-stabilising elements, Mo and V. In the subsequent annealing process, as the acicular martensite gradually decomposes to lamellar α phase, these elements will be diffused and ultimately form a lamellar α + β two-phase microstructure.

### 3.4. Mechanical Properties

#### 3.4.1. Tensile Properties

**Figure 7:**
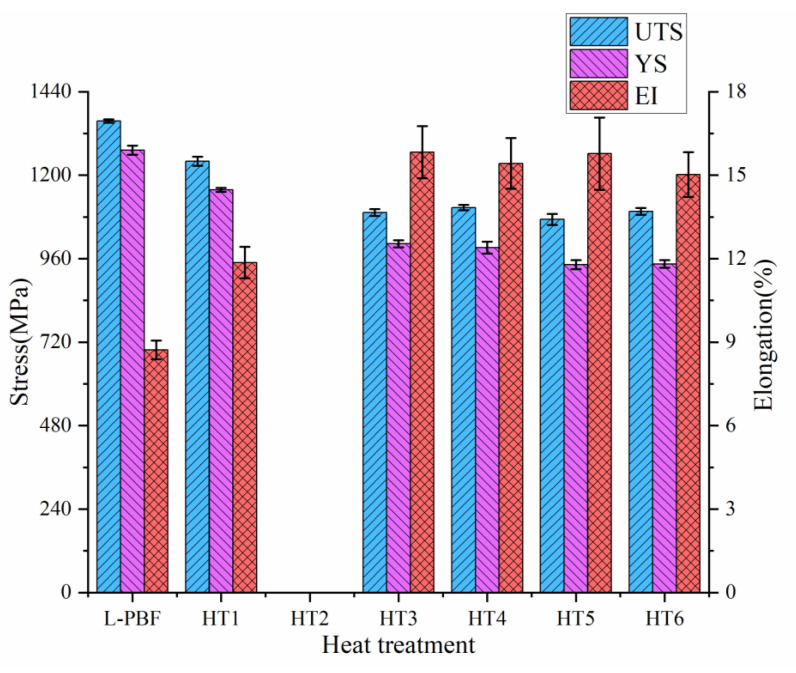
The mechanical properties of the as-built and annealing-treated samples. EI in the figure stands for the elongation at the break. Samples for HT2 built in a different batch in which building errors may present, which led to the observed premature failure.

The tensile properties of the L-PBF TA15 under different conditions are reported in Figure 7 and Table 4. Results suggest that the as-built samples exhibit extremely high ultimate tensile strength (UTS, 1356 MPa) and yield strength (1272 MPa), far exceeding the conventionally manufactured TA15 material [40,41]. However, the corresponding ductility is extremely poor, and the sample failed quickly after yielding without distinct necking, indicating poor deformability.

After the applied heat treatments, the ductility of the material has significantly improved, while the strength (both UTS and yield strength) has reduced by varying degrees. When the annealing temperature was increased from 630 °C to 950 °C, the strength tended to decrease at first and then stay steady; contrastingly, the elongation increased initially and then held steady. It can be seen that there is a trade-off between the strength and ductility of the L-PBF TA15 alloy. It can be attributed to a combination of several factors: (1) acicular martensite decomposition [8,14,27]; (2) grain coarsening [42]; (3) the appearance of secondary lamellar α in the transformed β matrix [13,21,22], such mechanism will be detail discussed in Section 4.2. Among all the conducted heat treatments, the most favourable mechanical properties can be achieved after annealing at 800–900 °C. Although the UTS is reduced to approximately 1100 Mpa, 18.9% reduced from as-built, the elongation reached around 12.5%, with a 79.08% improvement.

#### 3.4.2. Fracture Surface Analysis

**Figure 8:**
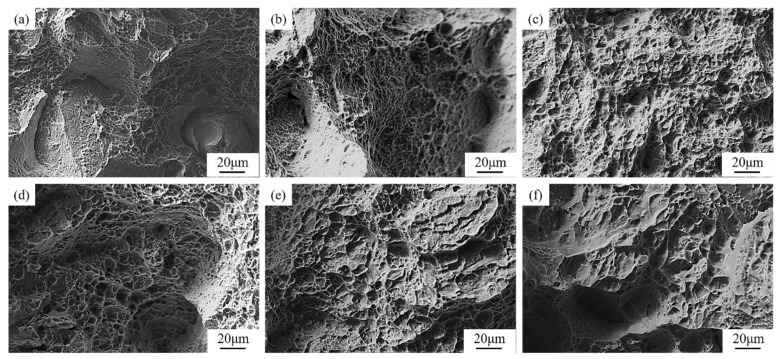
Tensile fracture surface of the as-built and heat-treated samples. (**a**) as-built; (**b**) 630 °C; (**c**) 800 °C; (**d**) 850 °C; (**e**) 900 °C; (**f**) 950 °C.

To further understand the tensile fracture behaviour of L-PBF TA15 before and after annealing, the fracture morphology of each condition is studied, as shown in Figure 8. The fracture surface of the as-built tensile (Figure 8a) is dominated by flat cleavage surfaces, cleavage steps and tear edges, with a few small and shallow dimples, which is a typical mixed brittle–ductile fracture. Figure 8b suggests that the fracture morphology of the TA15 sample after annealing (HT1) at 630 °C is quite similar to the as-built condition, mainly composed of intergranular fracture cleavage surfaces and small dimples. These two conditions showed similar mechanical properties, such as high strength and low ductility. Figure 8c–e are L-PBF TA15 after annealing at 800 °C (HT3), 850 °C (HT4) and 900 °C (HT5), respectively. Each of them shares very similar fracture morphologies. The surface is covered with small dimples, which is a typical sign of ductile fracture. As the annealing temperature increases, the dimple radiuses increase notably. When the annealing temperature increases to 950 °C (HT6), it can be noticed in Figure 8f that some brittle fractures reappear, accompanied by large size dimples. Correspondingly, a decrease of ductility has been observed in this condition.

## 4. Discussion

### 4.1. Microstructure Evolution during the Sub-Transus Heat Treatment

**Figure 9:**
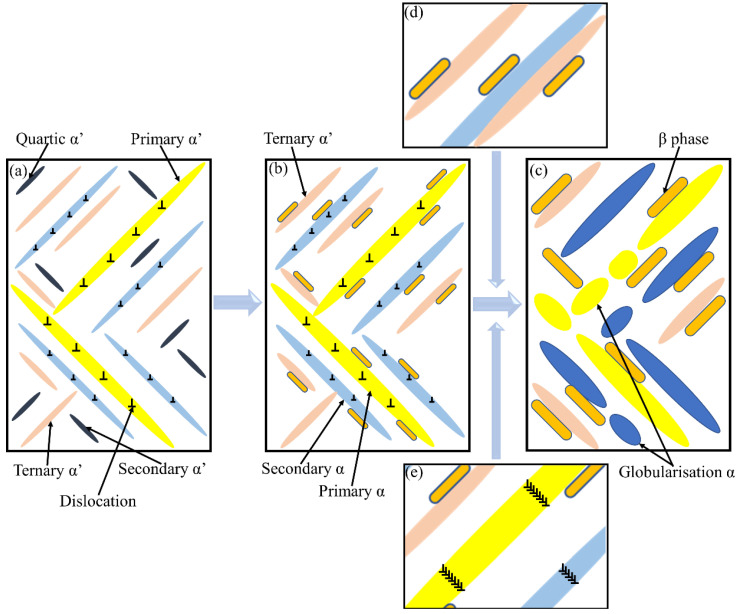
Schematic diagrams of the (**a**) α′ martensite hierarchical structure, (**b**) microstructure after low-temperature annealing and (**c**) microstructure after high-temperature annealing. (**d**) The coarsening and growth of the lamellar grains, (**e**) formation of early sub-grain boundaries.

In the presented study, the microstructural evolution of L-PBF TA15 is characterised via a series of heat treatments in which soaking temperatures are below the β-phase transus temperature. The mechanism that leads to the observed evolution is proposed and illustrated in Figure 9.

After heat treatment at 630 °C, fine martensite features can still be observed in the microstructure, which marks limited development in microstructure morphology compared with the as-built condition, as demonstrated in Figure 4a.

As the heating temperature elevates, α′ martensite starts to decompose, and recovery of α + β structure can be observed in Figure 4c (750 °C). Since α′ martensite is induced by diffusionless transformation, the hierarchical structure of the α′ martensite stores a large amount of crystal defects and retains enriched β stabiliser [36]. Therefore, the recovery of α-phase from α′ martensite during heat treatment can be approximately considered as a homogeneous nucleation process, which means a simultaneous transformation of primary α′, secondary α′ and ternary α′ to primary α, secondary α and ternary α, respectively, as indicated by the black arrows in Figure 9b. Moreover, during α phase recovery, crystal defects (e.g., dislocations) stored within the α′ phase not only will provide channels for solute diffusion but also will hinder the continuous growth of the α phase [17,20], which is conducive to the formation of refined lamellar α. Due to elemental diffusion, aluminium will concentrate in the hcp phase during the martensite decomposition, while vanadium will be expelled, as evidenced by the EDS line scan in Figure 6. Such element partitioning results in the nucleation of the α phase along the original α′ boundaries and the formation of the β phase at the newly formed α phase boundaries [20]. The inhomogeneous nucleation of the β phase mainly occurs at the grain boundaries of martensite grains, as well as the internal sub-grain boundary, as schematically illustrated in Figure 9b. Following the progressive precipitation of the β phase, the composition of the recovered α-phase approaches to a more equilibrium state [2,43]. Therefore, during the annealing, via grain nucleation and growth, the as-built metastable α′ phase directly decomposes to a near equilibrium α + β microstructure, as observed in Figure 4.

During the heat treatment, the adjacent lamellar α grains that share the same or similar orientation have a tendency to merge due to the Ostwald ripening phenomenon, thereby realising coarsening and growth of the lamellar grains [42]. When the heat treatment temperature approaches the β transus, the stored dislocations will be fully activated. The movement and annihilation of the isolated dislocation (as well as the partially connected dislocation network) will build up a series of dislocation arrays, and then polygonises to form the early sub-grain boundaries [21], as illustrated in Figure 9e. This is due to the fact that the stored energy in the dislocation network is higher than that of the dislocation array. According to the so-called low-energy dislocation structure theory proposed by Kuhlmann-Wilsdorf et al. [44], the dislocation network will spontaneously transform into the dislocation array to reduce the free energy. Therefore, the β phase may nucleate at newly formed sub-grains boundaries. The continuous growth of these β grains will split the original lamellar α grains into several fine α grains with reduced aspect ratio. The epitaxial growth of the split α grains will lead to the formation of nearly equiaxed α grains [21], as observed in Figure 5e, a phenomenon also known as the globularisation of α grain.

### 4.2. Microstructure-Mechanical Properties Relationship

Compared with the forged counterparts [40,41], the as-built L-PBF TA15 presents an approximately 30–40% higher ultimate tensile strength; however, its ductility is extremely poor, representing a significant mismatch between strength and ductility. Such a mismatch phenomenon is mainly attributed to the full acicular martensite microstructure in the as-built material. The slender grains of the acicular martensite provide a much more refined grain size of L-PBF TA15 than that of forged TA15. Refined grains are known to be a crucial factor for strengthening and hardening. Moreover, the martensite structure contains a high density of twins and dislocations [7,13,20]. The existing saturated lattice defects will resist the nucleation of new dislocations and hinder the formation of nano twin structures. Therefore, high density lattice defects provide additional material strengthening and promote the tendency for premature fracture [45]. Furthermore, the residual stress stored in the as-built material and the residual nano-β stripes within the acicular martensite will further limit the ductility of the material [46].

After HT1 (630 °C), only a limited amount of the acicular martensite starts to decompose, which can be evidenced by the presence of nano-size β precipitate, but the overall microstructure is still dominated by acicular martensite. After annealing, the internal residual stress of the material will be relieved, which results in the improvement of ductility and reduction of strength [8,13]. Under the conducted low-temperature annealing, the overall microstructure of the material does not change significantly. Therefore, compared with the as-built condition, the tensile properties and fracture morphology of these conditions are similar, and they both present a brittle-dominated feature.

After medium- and high-temperature annealing (750–900 °C, HT2-HT5), samples show similar microstructure with typical lamellar α + β two-phase structures. As the annealing temperature increases, the decomposing of martensite leads to an increase of the area fraction of recovered α + β, as well as a decrease of residual stress and density of lattice defects. This results in a sharp rise in plastic ductility, with the strength reduced by about 20% compared to the as-built sample. As shown in Figure 8c–e, although some relatively flat cracks are randomly distributed on the fracture surface, the sample displays an overall ductile failure. These conditions provide most dimple features consistent with the observed ductile behaviour. It is believed that some of the observed large dimples originated from the fracture of several adjacent α grains, whereas the tiny dimples are related to the failure of a single lamellar α grain [30].

When the annealing temperature further increases to 950 °C, a temperature close to the β-phase transus temperature, a large amount of β phases will be recovered from the as-built microstructure [13]. In the subsequent air cooling, a significant amount of secondary lamellar α phases will precipitate from the β phase and the transformed β matrix. Simultaneously, the lamellar α aspect ratio decreases significantly, due to the split of lamellar α and observed globularisation. The significant increase of the β phase is accompanied by a large number of fine secondary lamellar α distributed in the transformed β phase, forming a microstructure similar to the basket weave structure in localised areas. Such a feature can hinder crack propagation within the material, resulting in the formation of branches of single cracks, which helps to improve strength and toughness, while it is accompanied by a decrease of ductility [41,42]. The globularisation of the α and the significant coarsening of the primary lamellar α play a coordinating role in the deformation, so that the TA15 alloy can bear higher deformation and has higher ductility when it is strained; however, it is also accompanied by a significant loss of strength [21,22]. The combined influence of the two aspects causes a slight decrease of plastic ductility, as observed in this study. Furthermore, brittle fractures and larger dimples that appeared at the fracture surface can be considered a result of the precipitation of secondary layered α and the coarsening of crystal grains, as observed in Figure 8f.

This single heat-treatment study revealed that the microstructure significantly influences the mechanical properties of L-PBF TA15. Sun’s research on the heat treatment of traditional forged TA15 shows that a tri-modal microstructure can provide excellent comprehensive mechanical properties, ensuring high plastic ductility while maintaining high strength [28,40,41]. However, for L-PBF TA15 alloy, the difficulty lies in the formation of equiaxed α grains. Typically hot working deformation is required to obtain such equiaxed grains in the conventional manufacturing process, contrary to the advantage of near-net-shape shaping of L-PBF. This study reveals that using a heat treatment temperature close to the β-transus can successfully obtain equiaxed grains in L-PBF TA15. This information can be utilised to design a new heat treatment route for L-PBF TA15 alloy: after initial heat treatment at a sub-β-transus temperature to obtain equiaxed α grains, it is sensible to conduct subsequent heat treatments to tune the microstructure feature to achieve a more favourable tri-modal microstructure.

## 5. Conclusions

In this paper, the microstructure evolution of L-PBF TA15 under sub-transus heat treatment is investigated in detail, as well as its influence on tensile properties. The mechanism leads to the observed microstructure development, and the microstructure–mechanical properties relationship is discussed. The main findings are as follows:

1. The as-built L-PBF TA15 alloy exhibits a full acicular α′ martensite texture, which results in very high strength (1356 MPa) and hardness (395 HV), but low ductility (6.98%).
2. Martensite decomposition and recovery significantly influence the obtained microstructure after subsequent heat treatment. The α/α′ lamellar size remained nearly unchanged after a stress relief treatment at 630 °C/2 h. Then, a slow but steady increase of the lamellar thickness was observed with the increase of soaking temperature. The growth in lamella thickness becomes apparent only when the soaking temperature exceeds 900 °C.
3. After annealing, the L-PBF TA15 exhibited a fine basket-weave α + β structure. Material heat-treated between 800 and 900 °C contained fine lamellar α phase and some irregular β-phase precipitates, exhibiting the best ductility (~12.5%) and acceptable ultimate tensile stress (~1100 MPa). This can be attributed to the combined effects of the preferred phase fraction of the retained β and the relatively fine thickness of the retained α lath.
4. Globularisation of the α grain can be noticed when annealing at above 800 °C. This is linked to the subgrain boundary formation within the lamellar α and the subsequent grain splitting due to the β nucleation and growth.

## Figures and Tables

**Table 1:** The composition of the printed material, determined by EDS.

Composition	Ti	Al	Zr	Mo	V
wt%	Bal.	7.9%	2.2%	1.8%	2.0%

**Table 2:** Key temperature measured for the studied L-PBF TA15 and heat treatments applied in this study.

Sample Group	Temperature (°C)	Cooling Mode
Martensite start temperature (theoretical)	575	
HT1	630	AC
HT2	750	AC
Martensite finish temperature (theoretical)	800	
HT3	800	AC
HT4	850	AC
HT5	900	AC
HT6	950	AC
β tansus (measured)	1022	

**Table 3:** FHWM at different annealing temperatures (2θ ~40.5 °C) and the lattice constant of the β phase at various heat treatments.

Samples	FHWM	The Lattice Constant of β
L-PBF	0.42	
HT1 HT	0.34	3.224
HT2	0.28	3.227
HT3	0.28	3.233
HT4	0.28	3.235
HT5	0.31	3.244
HT6	0.3	3.248

**Table 4:** Measured mechanical properties of different samples.

Sample	σ_u_ (MPa)	σ_y_ (MPa)	ε (%)
L-PBF	1356 ± 5	1272 ± 13	6.98 ± 0.27
HT1	1240 ± 13	1158 ± 6	9.49 ± 0.45
HT2	Premature Failure	Premature Failure	Premature Failure
HT3	1093 ± 10	1003 ± 10	12.66 ± 0.75
HT4	1107 ± 8	992 ± 17	12.34 ± 0.73
HT5	1073 ± 16	943 ± 13	12.62 ± 1.04
HT6	1096 ± 10	945 ± 11	12.02 ± 0.64

## Data Availability

The data that support the findings of this study are available from the corresponding author, Sheng Zhang, upon reasonable request.
